# Long non-coding RNA Linc01133 promotes osteogenic differentiation of human periodontal ligament stem cells via microRNA-30c / bone gamma-carboxyglutamate protein axis

**DOI:** 10.1080/21655979.2022.2054912

**Published:** 2022-04-17

**Authors:** Qiang Li, Hangyu Zhou, Chuan Wang, Zhibin Zhu

**Affiliations:** a Stomatology Department, Chengdu Seventh People’s Hospital, Chengdu, Sichuan, China; b Plastic and maxillofacial surgery, Plastic and maxillofacial surgery, The Second Affiliated Hospital of Chongqing Medical University, Chongqing, China; c Maxillofacial surgery, Deyang stomatological hospital, Deyang, Sichuan, China

**Keywords:** Periodontitis, periodontal ligament stem cells, Linc01133, osteogenic differentiation, miR-30c, bone gamma-carboxyglutamate protein

## Abstract

Periodontitis is a chronic inflammation caused by the deposition of dental plaque on the tooth surface. Human periodontal ligament stem cells (hPDLSCs) have the potential of osteogenic differentiation. Long non-coding RNAs (lncRNAs) are collectively involved in periodontitis. This study was designed to explore the roles of Linc01133 in osteogenic differentiation of hPDLSCs. hPDLSCs obtained from the periodontal ligament (PDL) of patients with periodontitis were used to collect Linc01133, microRNA-30c (miR-30c), and bone gamma-carboxyglutamate protein (BGLAP) expression data, and their expression changes were traced during osteogenic differentiation of hPDLSCs. Quantitative reverse-transcription polymerase chain reaction as well as western blotting were used to analyze the levels of RNAs and proteins. Dual-luciferase reporter and RNA pull-down assays demonstrated the relationship between Linc01133, miR-30c, and BGLAP. Furthermore, alkaline phosphatase (ALP) staining and alizarin red staining were applied to evaluate the degree of osteogenic differentiation. Linc01133 was downregulated in the PDL of patients with periodontitis. Upregulated Linc01133 promoted osteogenic differentiation of hPDLSCs. Linc01133 could inhibit miR-30c expression by sponging miR-30c. miR-30c suppressed osteogenic differentiation. Additionally, miR-30c targeted BGLAP. Knockdown of BGLAP abrogated the effects of decreased miR-30c on osteogenic differentiation of hPDLSCs. Linc01133 acted as a ceRNA to regulate osteogenic differentiation of hPDLSCs via the miR-30c/BGLAP axis. Therefore, Linc01133 may participate in the progress of periodontitis.

## Graphical abstract

**Figure:**
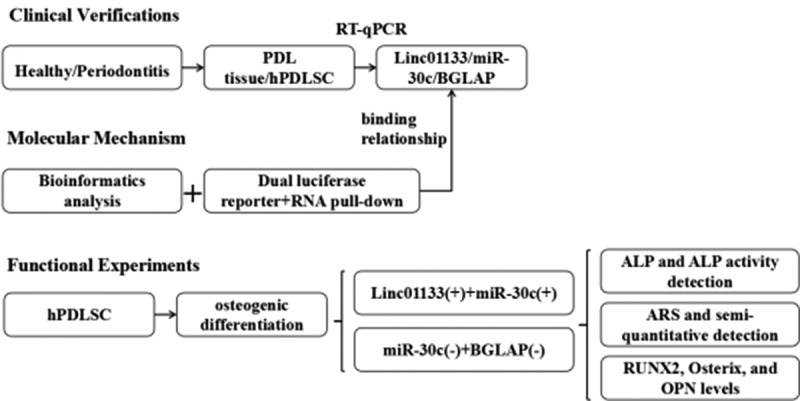

## Introduction

Periodontitis is an infectious disease of the periodontal tissue. Its main symptoms are loss of periodontal support (including that of the alveolar bone, cementum, and periodontal membrane) [1–3]. When the periodontal tissue becomes inflamed, there is an imbalance between the deposition of the alveolar bone and the resorption of the old bone, thereby inducing periodontitis [4].

The goal of periodontal therapy is to regenerate the damaged periodontal tissue, and repairing the alveolar bone defect is an important aspect of regeneration therapy [5]. At the cellular level, it is generally believed that osteoblasts and osteoclasts are two key cells that affect the bone remodeling process [6]. Human periodontal ligament stem cells (hPDLSC) have a high clonal proliferative ability and the ability to differentiate into osteoblasts, adipocytes, neuronlike cells, and chondroblasts under specific conditions [7]. Therefore, hPDLSC is regarded as a potential treatment modality for periodontitis in clinic.

Competing endogenous RNAs (ceRNAs) are transcripts that can regulate each other at post-transcription level by competing for shared miRNAs [8]. Long non-coding RNAs (lncRNAs), which function as the ceRNA by sponge microRNAs (miRNAs) to regulate diverse disorders have been reported over the last decade [9]. LncRNAs have been reported to modulate various diseases, including periodontitis [10–13]. Linc01133 is an emerging tumor-associated lncRNA [14,15], and Tang et al. found it to be abnormally suppressed in periodontitis [16]. However, the underlying molecular mechanisms of Linc01133 in hPDLSCs require further elucidation.

The miR-30 family was found to negatively regulate the osteogenic differentiation [17–19]. Wu et al. suggested miR-30c is a key negative regulator of bone morphogenetic protein (BMP)-induced osteogenic differentiation of MC3T3-E1 cells [17]. BGLAP is a small molecule of protein produced by osteoblasts, odontoblasts, and odontoblasts and is the most abundant non-collagen protein in bone-like tissue [20,21]. BGLAP is considered the most characteristic phenotypic marker of osteoblasts, and its protein product is a large amount of non-collagenous protein secreted at the beginning of mineralization after cell proliferation [22,23].

In the present study, we speculated that Linc01133 may bind with miR-30c to modulate BGLAP expression to regulate osteogenic differentiation ability of hPDLSCs. Our study aimed to investigate the molecular mechanisms of Linc01133 in the development of periodontitis.

## Materials and methods

### hPDLSCs

The process of obtaining, isolating, and cultivating hPDLSCs was approved by the Chengdu Seventh People’s Hospital Ethics Committee (2,015,015), and informed consent was obtained from the patients. Thirty periodontitis patients (18 male, 12 female), mean age 36 (20 to 57), with alveolar bone absorption and tooth loosening who need tooth extraction were obtained in this study according to 2017 World Workshop on the classification of periodontitis, and thirty healthy volunteers who had teeth removed for orthodontic purposes were obtained in this study. After the root surface was rinsed repeatedly with phosphate buffer saline (PBS) solution, 1/3 of the periodontal ligament (PDL) tissue in the root was scraped and clipped into small tissue blocks. After centrifugation at 1,000 rpm, the supernatant was discarded. The precipitate was digested with a mixture of 3 mg/mL type I collagenase (Sigma-Aldrich) and 4 mg/mL dispase (Sigma-Aldrich). After centrifugation, the supernatant was added into α-MEM (Hyclone) culture medium containing 20% fetal bovine serum (FBS; Invitrogen) and 1% penicillin/streptomycin (Hyclone, USA), and cultured in a 5% CO_2_ incubator at 37°C for two weeks. The solution was changed 4 days later, and then changed every 3 days. When the cells were filled to about 70% to 80% of the bottom of the culture flask, digestion and passage were carried out with 0.25% trypsin (Invitrogen). Cells were digested and single cell-derived hPDLSC colony cultures were obtained. The first-generation hPDLSCs were inoculated into 96-well plates at 100 μl/well with a density of 10 ~ 15 cells/mL by limited dilution method [24]. After 12 h, the holes containing only a single cell were marked, and when the cells grew to 1/2 of the area at the bottom of the hole, the culture was extended. The fifth-generation cells were selected for subsequent experiments.

### Cell transfection

Linc01133 overexpression vector (Linc01133) and its empty vector (vector), upregulated miR-30c plasmids (Mimic) and negative control (NC) mimic, miR-30c inhibitor (inhibitor) and NC inhibitor and suppressed bone gamma-carboxyglutamate protein (BGLAP) (Si-BGLAP 1#, Si-BGLAP 2#) and Si-nc were obtained from GenePharma (Shanghai, China) [25]. The hPDLSCs were inoculated on 6-well plates to evenly distribute the cells. After the cells adhered to the wall, the culture medium without double antibodies containing 10% FBS (Invitrogen) was changed until the cell density reached about 60% for transfection. Then, 10 μL plasmids were dissolved in 500 μL serum-free medium per well at room temperature for 5 min, and 20 μl Lipofectamine 3000 (Invitrogen) was dissolved in 500 μL serum-free medium from each well and stood at room temperature for 30 min. The mixture was added into the well plate to complete the transfection. The transfection efficiency was then evaluated by quantitative reverse-transcription polymerase chain reaction (qRT-PCR) at 24 h after transfection.

### Osteogenic differentiation

The fifth-generation hPDLSCs reached 80% confluence in 6-well plates. They were incubated with osteogenic medium (100 nM dexamethasone, 50 mg/ml ascorbic acid, and 5 mM b-glycerophosphate; Sigma-Aldrich) for 10 to 14 days, and the medium was changed every 3 days [26]. Total protein or RNA from hPDLSCs (5 × 10^5^ cells) was extracted after osteogenic differentiation.

### Alkaline phosphatase (ALP) staining and ALP activity detection

The hPDLSC suspension was added to the petri dish at day 14 of osteogenic induction. The cells were then rinsed with PBS and fixed with cold propanol (Solarbio) for 10 min. The incubation solution (3% β-glycerophosphate, 2% sodium barbiturate, 2% CaC1_2_, 2% MgSO_4_) was then added at 37°C and kept for 4–6 h. The petri dish was soaked in 2% cobalt nitrate for 3–5 min and washed with distilled water several times [27]. Finally, 1% ammonium sulfide (Solarbio) was added and washed with running water after 2 min. The reaction was observed under an inverted microscope (Nikon) and photographed. Then ALP activity was determined according to the kit instructions (Nanjing Jiancheng Biological Co., LTD). The optical density of samples in each group was measured using a spectrophotometer (Nikon) at 520 nm wavelength, and ALP activity of each group was calculated.

### Alizarin red staining (ARS) and semi-quantitative detection

hPDLSCs were cultured in culture medium for 21 days, fixed with 4% paraformaldehyde (Solarbio) for 30 min, then stained with 0.5% alizarin red (pH = 8.8) at room temperature for 1 h, and observed and photographed under an inverted microscope (Nikon). After the supernatant was discarded, 100 mmol/L cetylpyridine chloride was added after washing the sample three times in PBS and incubated for 1 h at room temperature. The A value of 562 nm wave long wavelength samples was measured by ultraviolet spectrophotometry, and the test was repeated three times for each sample.

### qRT-PCR

The RNA of PDL tissues, as well as of hPDLSCs, before and after osteogenic induction was extracted by Trizol regeant (Invitrogen). The concentration of the RNA was determined, and the RNA was reversely transcribed into cDNA (SuperScript First-Strand Synthesis Kit; Invitrogen). The cDNA obtained through reverse transcription was detected by fluorescence quantitative PCR using Syber Green fluorescence quantitative PCR kit (Takara) [27]. The relative expression levels of mRNA and miRNA were calculated by 2-^ΔΔCt^ method. Primer sequence (5’3’): Linc01133, forward: TGGTGGAGAGAATGGAGG, reverse: AACCCAGTTCCTTAGAATCTTC; runt-related transcription factor 2 (RUNX2), forward: CCCGTGGCCTTCAAGzGT, reverse: CGTTACCCGCCATGACAGTA; Osterix, forward: ACCCACCTCAGGCTATGCTA, reverse: TGCCCCCATATCCACCACTA; osteopontin (OPN), forward: AGAGCTAGTTTGCCTGCGTT, reverse: AAGCACATGTTTGCCAGCAG; miR-30c, forward: GTCGTATCCAGTGCAGGGTCCGAGGTATTCGCACTGGATACGACGCTGA, reverse: GCCGCTGTAAACATCCTACACT; BGLAP, forward: ACCAGGCTCCCTTTCCTTTG, reverse: TCAGCCAACTCGTCACAGTC; glyceraldehyde-3-phosphate (GAPDH): forward: AAGGTGAAGGTCGGAGTCA, reverse: GGAAGATGGTGATGGGATTT; and U6, forward: CCAAATCTAGCTGCTGCGGT, reverse: CAACAGGCTCGTGAAAGACC. The reaction conditions were as follows: 95°C degeneration, 20 min; 95°C, 30s; and 60°C, 1 min for 40 cycles. The results were automatically calculated by the software according to the standard curve. To verify the reproducibility and amplification efficiency of the method, the experiment was repeated three times.

### Western blotting assay

After 14 days of osteogenic induction, the total protein of hPDLSCs in each group was extracted, and the protein expression levels of osteogenic differentiation markers RUNX2, Osterix, and OPN were detected [28]. In simple terms, the osteogenic induced hPDLSCs were added with RIPA lysate (Solarbio) to extract total protein, and the protein concentration was determined using a BCA kit (Nanjing Kaiji Biological Co., LTD). According to the molecular weight of the target protein and the specification requirements of 10% separation glue, 40 μL protein was added to each well, and 5 μL protein marker was added to the adjacent well of the protein sample. Then, electrophoresis was performed at 60 V constant pressure for 30 min and 160 V constant pressure for 1 h. The glue was then cut and transferred to a PVDF membrane (Millipore) of the same size at 100 V constant pressure. Then the protein was sealed, the primary antibody diluent was added for overnight incubation (4°C), and the second antibody diluent was transferred to room temperature for incubation for 1 h on the next day. Finally, ECL luminescent reagent (Bosch Bio) was used to color and fix PVDF membrane on gel imager.

### Binding sites prediction

Bioinformatics websites StarBase (https://starbase.sysu.edu.cn/) as well as TargetScan [29] (http://www.targetscan.org) were used for predicting binding sites between miR-30c and Linc01133 or BGLAP.

### Dual luciferase reporter assay

The wild type (wt) and mutant type (mut) 3’-UTR region of LNC01133, as well as BGLAP luciferase reporter vectors, were designed and synthesized by RiboBio. Linc01133 3’-UTR (wt and mut) or BGLAP 3’-UTR (wt or mut) vectors containing binding sites with miR-30c were inserted into the firefly luciferase gene, and then co-transfected into hPDLSCs of each group under the manufacturer instructions provided for Lipofectamine 3000 (Invitrogen) [29].

### RNA pull-down assay

We combined 500 μg streptavidin magnetic beads with 200 pmol biotin-labeled miR-30c mimic and added into total RNA extracted from hPDLSCs of each group [29]. The pulled RNA complex was collected after adding the eluting buffer at room temperature and incubating for 30 min. Finally, the Linc01133 and BGLAP levels were quantitatively analyzed by qRT-PCR.

### Statistical analysis

SPSS version17.0 statistical software was used for statistical analysis, and all data were expressed as mean ± standard deviation. Student’s t-test (two groups) and one-way ANOVA (multiple groups) followed by Tukey’s test were applied for difference analysis. Differences with *p* < 0.05 were regarded statistically significant.

## Results

### Linc01133 expression level was positively related with osteogenic differentiation of hPDLSC

**Figure 1.**
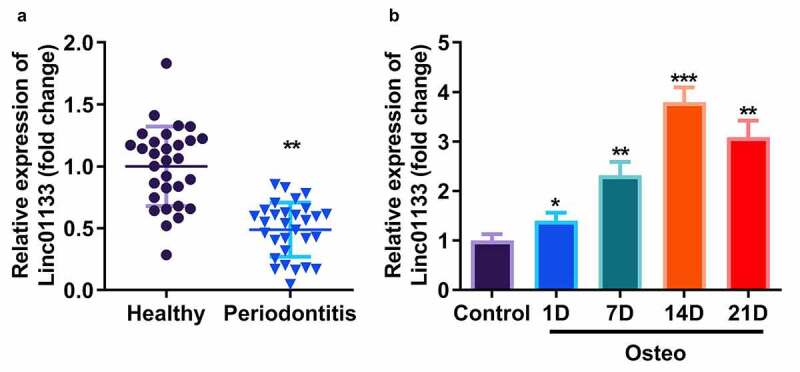
Linc01133 expression level was positively related with osteogenic differentiation of hPDLSC. (a)qRT-PCR was used to evaluate the expression level of Linc01133 in PDL tissues derived from periodontitis patients with periodontitis as well as healthy controls. (b) qRT-PCR was used to detect the expression level of Linc01133 in hPDLSCs derived from periodontitis patients with periodontitis before and after osteogenesis induction. *p < 0.05, **p < 0.01, ***p < 0.001. vs. healthy, and control group. qRT-PCR, quantitative reverse-transcription polymerase chain reaction; hPDLSC, human periodontal ligament stem cells; ARS, alizarin red staining.

gLinc01133 mRNA expression was evaluated in PDL tissues and hPDLSCs. Linc01133 expression was dramatically lower in PDL tissues derived from the periodontitis group than that in the healthy group (Figure 1a). The expression of Linc01133 was positively associated with the incubation time of hPDLSCs in osteogenic medium and peaked on day 14 (Figure 1b).

### Upregulation of Linc01133 contributed to osteogenic differentiation of hPDLSC

**Figure 2.**
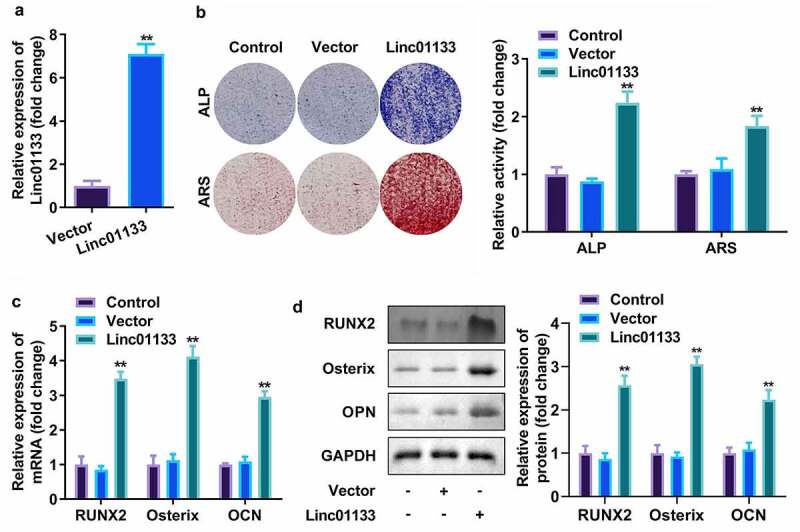
Upregulation of Linc01133 contributed to osteogenic differentiation of hPDLSCs. (a)qRT-PCR assay was applied to confirm the transfection efficiency of Linc01133. (b) ALP staining and ARS staining of hPDLSC on day 14 in osteogenic medium. (c) qRT-PCR analysis for the expression of RUNX2, Osterix, and OPN. (d) Western blotting analysis for the expression of RUNX2, Osterix, and OPN. **p < 0.01, vs. vector group. qRT-PCR, quantitative reverse-transcription polymerase chain reaction; hPDLSC, human periodontal ligament stem cells; ALP, alkaline phosphatase; ARS, alizarin red staining.

Then whether Linc01133 could modulate osteogenic differentiation of hPDLSC was investigated. qRT-PCR analyses of Linc01133 expression in hPDLSCs revealed that hPDLSCs were successfully transfected with overexpressed Linc01133 vector (Figure 2a). At the same time, the number of ALP and ARS staining mineralized nodules in the overexpression Linc01133 group was notably more than that in the control group and empty plasmid transfection group (Figure 2b). The semi-quantitative results also showed that the activity of ALP and ARS was greatly increased by overexpressed Linc01133 (Figure 2b). Moreover, as markers of osteogenic differentiation, the secretion of RUNX2, Osterix, and OPN was markedly increased in upregulated Linc01133 osteogenic induced hPDLSCs and was reflected in both protein and mRNA levels (Figure 2c,d).

### Linc01133 targeted miR-30c and repressed its expression

**Figure 3.**
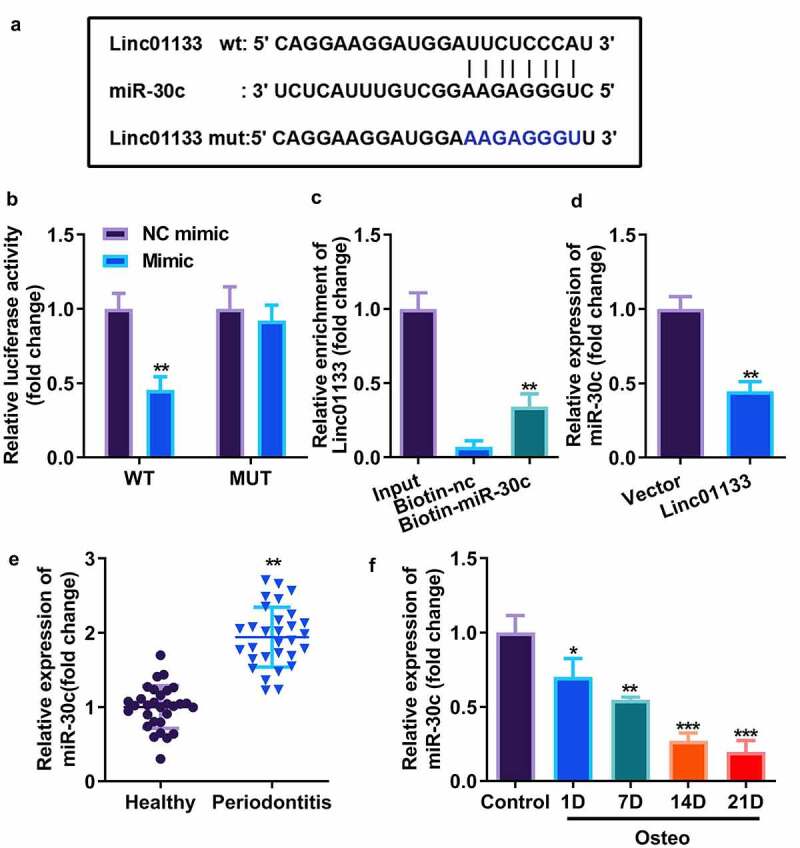
Linc01133 targeted miR-30c and repressed its expression. (a)Predicted binding sites of miR-30c in the 3’-UTR of wt Linc01133 (b) Luciferase activity of hPDLSCs transfected with miR-30c mimic in the wt Linc01133 as well as mut Linc01133 groups. (c) Lysates from hPDLSCs with miR-30c overexpression were subject to biotinylated miR-30c pull-down assay, and the expression levels of Linc01133 were detected by qRT-PCR. (d) miR-30c expression in hPDLSCs transfected with overexpressed Linc01133 vector. (e) qRT-PCR was used to evaluate the expression level of miR-30c in PDL tissues derived from periodontitis patients with periodontitis as well as healthy controls. (f) qRT-PCR was used to detect the expression level of miR-30c in hPDLSCs derived from patients with periodontitis patients before and after osteogenesis induction. *p < 0.05, **p < 0.01, ***p < 0.001. vs. nc mimic, biotin-nc, vector, healthy and control group. qRT-PCR, quantitative reverse-transcription polymerase chain reaction; hPDLSC, human periodontal ligament stem cells; PDL, periodontal ligament.

Bioinformatics analysis showed that the 587–608 bases of Linc01133 may be the binding site of miR-30c (Figure 3a). The activity of luciferase was obviously decreased after transfection of wt Linc01133 3’-UTR vector and miR-30c mimic (Figure 3b). The interaction between the two was further confirmed by the RNA pull-down experiment, which was carried out by designing a biotin-labeled miR-30c mimic and by enriching and pulling down RNA with magnetic beads. The qRT-PCR results confirmed that the miR-30c mimic could specifically bind to Linc01133 compared with unrelated control probe (Figure 3c). Furthermore, miR-30c was markedly suppressed by Linc01133, suggesting that miR-30c could be sponged by Linc01133 (Figure 3d). qRT-PCR results confirmed that miR-30c expression increased in PDL tissues derived from the periodontitis group and decreased with the time lapse of osteogenic induction of hPDLSCs (Figure 3e,f).

### Overexpression of miR-30c reversed the effects of Linc01133 on osteogenic differentiation of hPDLSC

**Figure 4.**
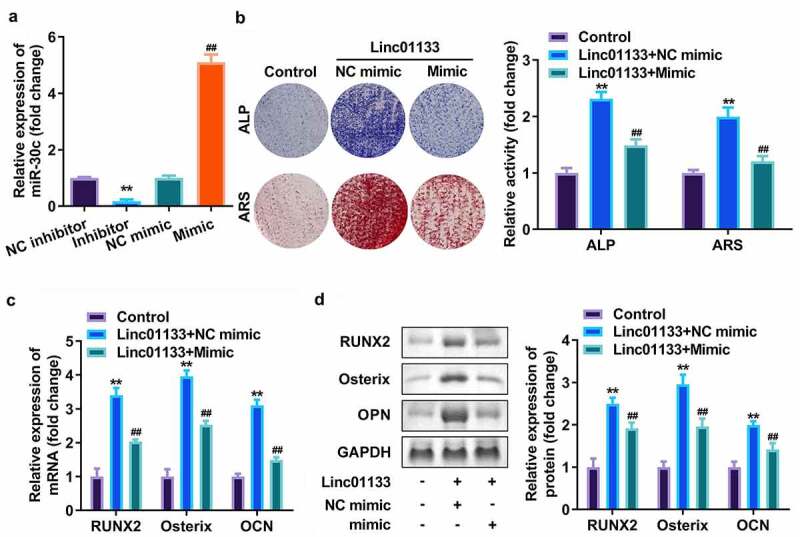
Overexpression of miR-30c reversed the effects of Linc01133 on osteogenic differentiation of hPDLSC. (a)The miR-30c expression levels were detected by qRT-PCR. (b) Images and quantification of ALP staining and ARS of hPDLSCs transfected with Linc01133 and miR-30c mimic. (c) qRT-PCR analysis for the expression of RUNX2, Osterix, and OPN. (d) Western blotting analysis for the expression of RUNX2, Osterix, and OPN. **p < 0.01, vs. nc inhibitor, and control group. ##*p* < 0.01, vs. nc mimic, and Linc01133+ nc mimic group. qRT-PCR, quantitative reverse-transcription polymerase chain reaction; hPDLSC, human periodontal ligament stem cells.

Subsequently, miR-30c was upregulated in hPDLSCs to verify its effect on Linc01133. qRT-PCR analyses indicated that miR-30c expression level was dramatically upregulated after transfection with miR-30c mimics but downregulated on miR-30c inhibitor addition (Figure 4a). ALP as well as ARS staining assays showed that miR-30c overexpression reduced mineralized nodules induced by Linc01133 (Figure 4b). Moreover, upregulation of miR-30c notably inhibited mRNA and protein expression of RUNX2, Osterix, and OPN, which had been elevated by Linc01133 (Figure 4c,d).

### miR-30c directly targeted BGLAP in hPDLSCs

**Figure 5.**
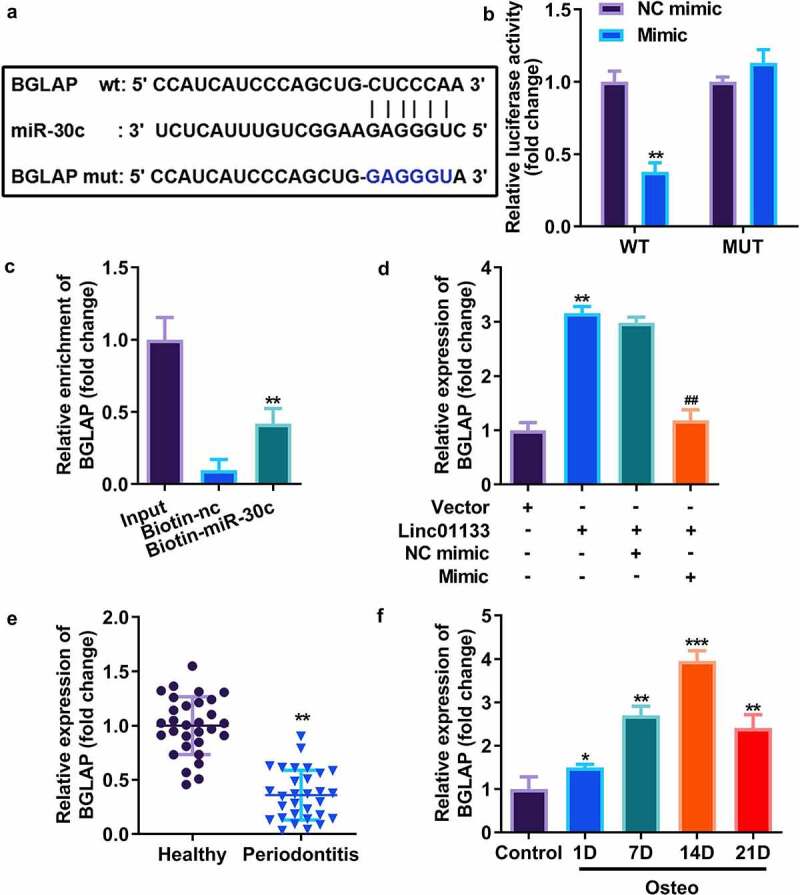
miR-30c directly targeted BGLAP in hPDLSCs. (a)Predicted binding sites of miR-30c in the 3’-UTR of wt BGLAP (b) Luciferase activity of hPDLSCs transfected with miR-30c mimic in the wt BGLAP as well as mut BGLAP groups. (c) Lysates from hPDLSCs with miR-30c overexpression were subject to biotinylated miR-30c pull-down assay, and the expression levels of BGLAP were detected by qRT-PCR. (d) BGLAP expression in hPDLSCs transfected with overexpressed Linc01133 vector and/or miR-30c mimic. (e) qRT-PCR was used to evaluate the expression level of BGLAP in PDL tissues derived from periodontitis patients with periodontitis as well as healthy controls. (f) qRT-PCR was used to the detect expression level of BGLAP in hPDLSCs derived from periodontitis patients with periodontitis before and after osteogenesis induction. *p < 0.05, **p < 0.01, ***p < 0.001. vs. nc mimic, vector, biotin-nc, healthy and control group. ##*p* < 0.01, vs. Linc01133+ nc mimic group. qRT-PCR, quantitative reverse-transcription polymerase chain reaction; hPDLSC, human periodontal ligament stem cells; PDL, periodontal ligament; BGLAP, bone gamma-carboxyglutamate protein; wt, wild type; mut, mutant type.

Downstream genes of miR-30c were selected by TargetScan, and the results showed that miR-30c potentially bound to BGLAP (Figure 5a). Dual luciferase reporter assay indicated that luciferase activity was remarkably decreased in cells co-transfected with wt-BGLAP and miR-30c mimic (Figure 5b). RNA pull-down assay further indicated that miR-30c could directly bind to 3’-UTR of BGLAP (Figure 5c). To investigate the binding relationship thoroughly, the expression of BGLAP in hPDLSCs transfected with miR-30c and/or Linc01133 was measured, and the results indicated that miR-30c mimics markedly reduced the expression of BGLAP while Linc01133 notably upregulated it (Figure 5d). Our findings suggest that BGLAP expression was decreased in PDL tissues derived from the periodontitis group and positively correlated with the incubation time of hPDLSCs in osteogenic medium and peaked on day 14 (Figure 5e,f).

### miR-30c targeted BGLAP to affect osteogenic differentiation of hPDLSC

**Figure 6.**
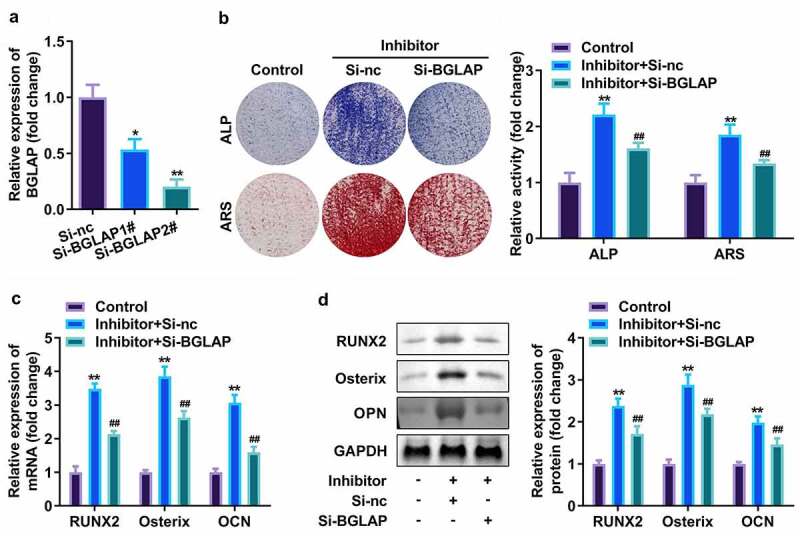
miR-30c targeted BGLAP to affect osteogenic differentiation of hPDLSC. (a)qRT-PCR assay was applied to confirm the transfection efficiency of BGLAP. (b) ALP staining and ARS staining of hPDLSC on day 14 in osteogenic medium. (c) qRT-PCR analysis for the expression of RUNX2, Osterix, and OPN. (d) Western blotting analysis for the expression of RUNX2, Osterix, and OPN. *p < 0.05, **p < 0.01, vs. si-nc, and control group. ##*p* < 0.01, vs. inhibitor+si-nc group. qRT-PCR, quantitative reverse-transcription polymerase chain reaction; hPDLSC, human periodontal ligament stem cells; ALP, alkaline phosphatase; ARS, alizarin red staining.

Finally, the effect of BGLAP was accessed. As showed in Figure 6a, BGLAP expression was dramatically decreased after interference RNA of BGLAP was transfected into hPDLSCs, which was more potent in the si-BGLAP 2# group (Figure 6a). Furthermore, APL and ARS activity as well as expression level of RUNX2, Osterix, along with OPN were markedly increased by suppressed miR-30c (Figure 6b); however, downregulated BGLAP then abrogated these effects of miR-30c inhibitor on hPDLSCs (Figure 6c,d).

## Discussion

In the present study, we unveiled the underlying mechanisms that Linc01133 contributed to the osteogenic differentiation of hPDLSCs by sponging miR-30c to regulate expression of BGLAP gene. Together, overexpression of Linc01133 promoted the osteogenic differentiation of hPDLSCs by modulating the miR-30c/BGLAP axis.

Periodontitis is characterized by severe destruction of periodontal supporting tissues, such as of the gingiva and alveolar bone [1,2]. In recent years, many lncRNAs play an important regulatory role in the occurrence and development of periodontitis [10–12]. Li et al. found 3 lncRNAs that might participate in the lncRNA-associated ceRNA network in periodontitis [30], which was in keeping with the results of Wang et al. [31]. Furthermore, the expression of RNA-binding protein Lin28A was positively correlated with the osteogenic differentiation of hPDLSCs after induction, and Lin28A contains multiple binding sites of lncRNA TUGl, which is a potential target for TUGl regulation of osteogenic differentiation [32]. Meanwhile, lncRNA can also regulate the expression of osteogenic genes through competitive binding of transcription factors. For example, lncRNA HOXA-AS3 directly regulates the activity of H3K27me3 on the osteogenic transcription factor RUNX2, thereby inhibiting osteogenic differentiation of bone marrow stromal cells [32]. Therefore, we speculated that lncRNA could affect osteogenic differentiation of hPDLSCs and thereby regulate the process of periodontitis.

Linc01133 is a newly identified lncRNA in periodontitis through bioinformatics [16]. Our data suggested that Linc01133 was abnormally decreased in periodontitis compared with the control group, and its expression was positively correlated with the osteogenic induction time of hPDLSCs. In this study, ALP and ARS staining indicated that upregulation of Linc01133 promoted osteogenic differentiation of hPDLSCs in *vitro*. At the same time, qRT-PCR and Western blot analysis confirmed this at the RNA and protein levels of osteogenic-related proteins. Thence, ou findings demonstrated the potential regulatory role of Linc01133 on osteogenic differentiation of periodontitis.

Interestingly, we found that 3’-UTR Linc01133 could bind to miR-30c and function as miR-30c sponge. Meanwhile, miR-30c was elevated in periodontitis clinical samples and negatively regulated by the osteogenic induction time of hPDLSCs. Besides, we also observed that upregulated miR-30c promoted osteogenic differentiation. These findings suggested that Linc01133 promotes the osteogenic differentiation of hPDLSCs by negatively regulating miR-30c, which was in line with the results of previous studies [17].

It has been confirmed that hPDLSCs have typical osteoblast characteristics and can synthesize BGLAP [33]. In this research, BGLAP was verified to be a target gene of miR-30c; it was suppressed in PDL tissue and upregulated in osteogenesis-induced hPDLSCs. Furthermore, suppressed BGLAP alleviated effects of suppressed miR-30c on osteogenic differentiation of hPDLSCs.

## Conclusion

Our research suggests that Linc01133 acted as ceRNA to regulate osteogenic differentiation of hPDLSCs via the miR-30c/BGLAP axis. It might be possible to support periodontal regeneration by upregulating Linc01133.

## Data Availability

Not applicable.
